# Sustained Hemodynamic and Clinical Improvements for a Patient With Idiopathic Pulmonary Arterial Hypertension Over 1.5 Years After Balloon Atrial Septostomy: A Case Report

**DOI:** 10.3389/fcvm.2022.868123

**Published:** 2022-04-07

**Authors:** Kae-Woei Liang, Kuo-Yang Wang

**Affiliations:** ^1^Cardiovascular Center, Taichung Veterans General Hospital, Taichung, Taiwan; ^2^School of Medicine, National Yang Ming Chiao Tung University, Taipei, Taiwan; ^3^School of Medicine and School of Life Science, National Chung Hsing University, Taichung, Taiwan; ^4^Center for Pulmonary Arterial Hypertension and Pulmonary Vascular Disease, China Medical University Hospital, Taichung, Taiwan

**Keywords:** balloon atrial septostomy (BAS), idiopathic pulmonary arterial hypertension (IPAH), intracardiac echocardiography (ICE), prostacyclin analogs, case report

## Abstract

Balloon atrial septostomy (BAS) is an indicated treatment for subjects with idiopathic pulmonary arterial hypertension (IPAH), particularly for those with advanced right heart failure before bridging to lung transplantation. The mid-term clinical and hemodynamic benefits of BAS are not well studied. Here, we present a young female patient with IPAH who received maximal target medication and was admitted to our hospital due to advanced right heart failure. She had transition of subcutaneous to intravenous (IV) prostacyclin analogs (PA) injection and was registered for lung transplantation. The baseline mean right atrium (RA) pressure was 14 mmHg. BAS was performed with a balloon of 6 mm under intracardiac echocardiography (ICE) guidance. Systemic cardiac output (CO) (2.9–3.5 L/min) and oxygen delivery (OD) (291–318 ml/min) both increased after the BAS. Right heart failure was alleviated to function class II. One and a half years later, she received cardiac catheterization again. The second baseline mean RA pressure was 5 mmHg, left atrium (LA) pressure was 2 mmHg, and systemic CO was 3.3 L/min. These data indicated sustained hemodynamic improvements. The second course of BAS was performed under ICE guidance with a balloon of 8 mm. After the second BAS, her RA pressure was 3 mmHg, LA pressure was 3 mmHg, and CO was 3.4 L/min. In conclusion, BAS and IV PA infusion were effective in maintaining mid-term hemodynamic benefits and in stabilizing the critical right heart failure in a patient with IPAH over a 1.5-year period.

## Introduction

Balloon atrial septostomy (BAS) in an indicated (class IIb) treatment option for patients with idiopathic pulmonary arterial hypertension (IPAH) with concurrent advanced right heart failure before bridging to lung transplantation (1). Lung transplantation has a higher mortality in IPAH compared to other conditions and is unavailable for majority of patients in many parts of the world (2). Recent meta-analysis confirmed immediate hemodynamic benefits of BAS, including reduction of right atrium (RA) pressure and increases of systemic cardiac output (CO) and oxygen delivery (OD) (3). However, its mid-term clinical and hemodynamic benefits are rarely reported. Herein, we present a young female with IPAH having received maximal target medication and was admitted due to advanced right heart failure. She received conversion of continuous infusion of prostacyclin analogs (PA) from subcutaneous to intravenous (IV) route plus BAS to rescue her from severe right heart failure episode. One and a half years later, hemodynamic data from cardiac catheterization confirmed both well maintained reduction of RA pressure and increases of systemic CO and OD.

## Case Presentation

**Figure 1:**
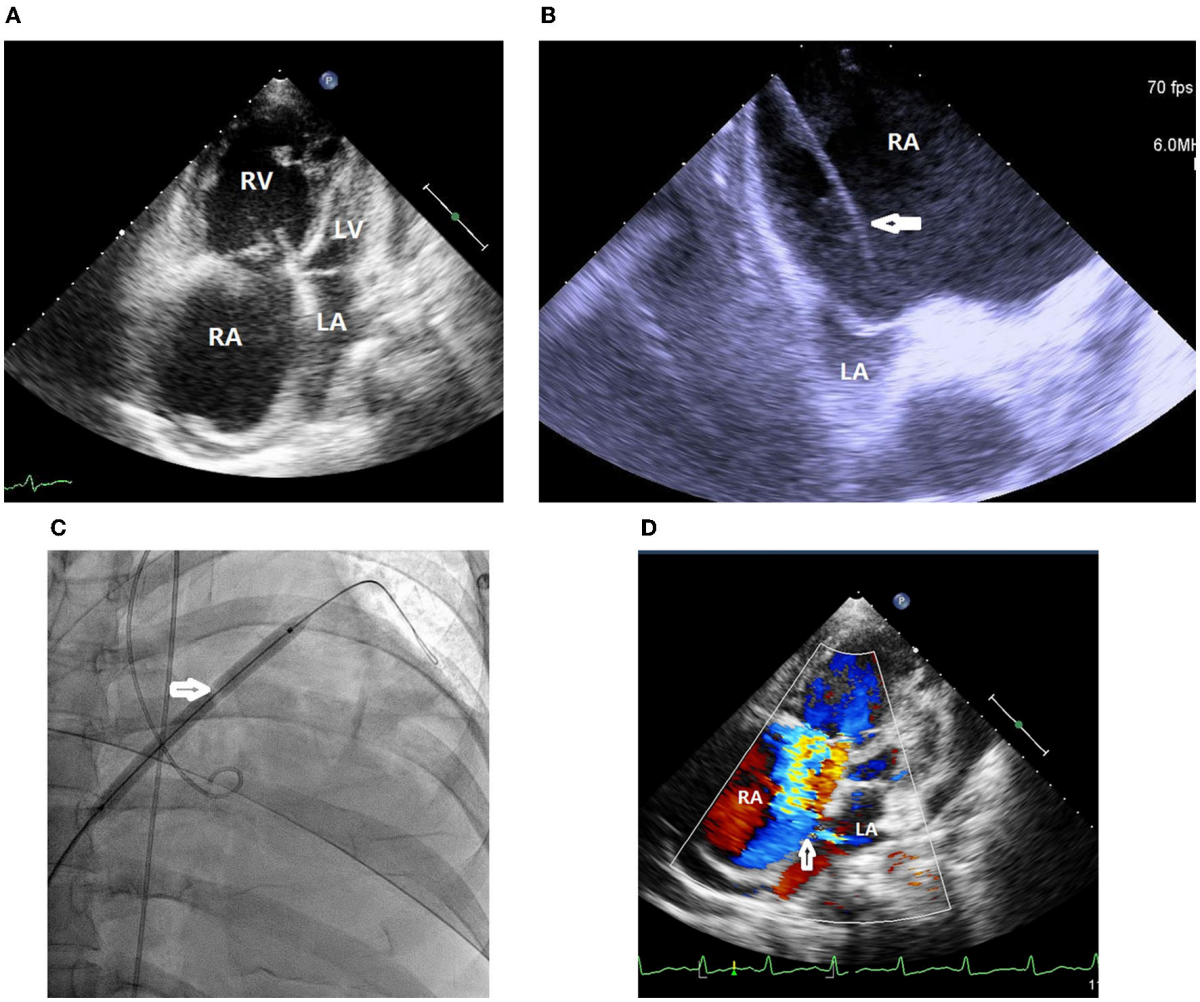
**(A)** Trans-thoracic echocardiogram revealed marked dilated right atrium and right ventricle in addition to small and compressed left atrium and left ventricle. RA, right atrium; RV, right ventricle; LA, left atrium; LV, left ventricle. **(B)** Intracardiac echocardiogram (ICE) showed marked dilated right atrium and small compressed left atrium. A transseptal Brockenbrough needle and a Mullins sheath (white arrow) was probing the inter-atrial septum with a tenting sign. RA, right atrium; LA, left atrium. **(C)** The atrial septum was dilated with a 6 mm × 8 cm balloon (white arrow). **(D)** Transthoracic echocardiogram confirmed the establishment of an inter-atrial shunt from right to left (white arrow). RA, right atrium; LA, left atrium.

**Figure 2:**
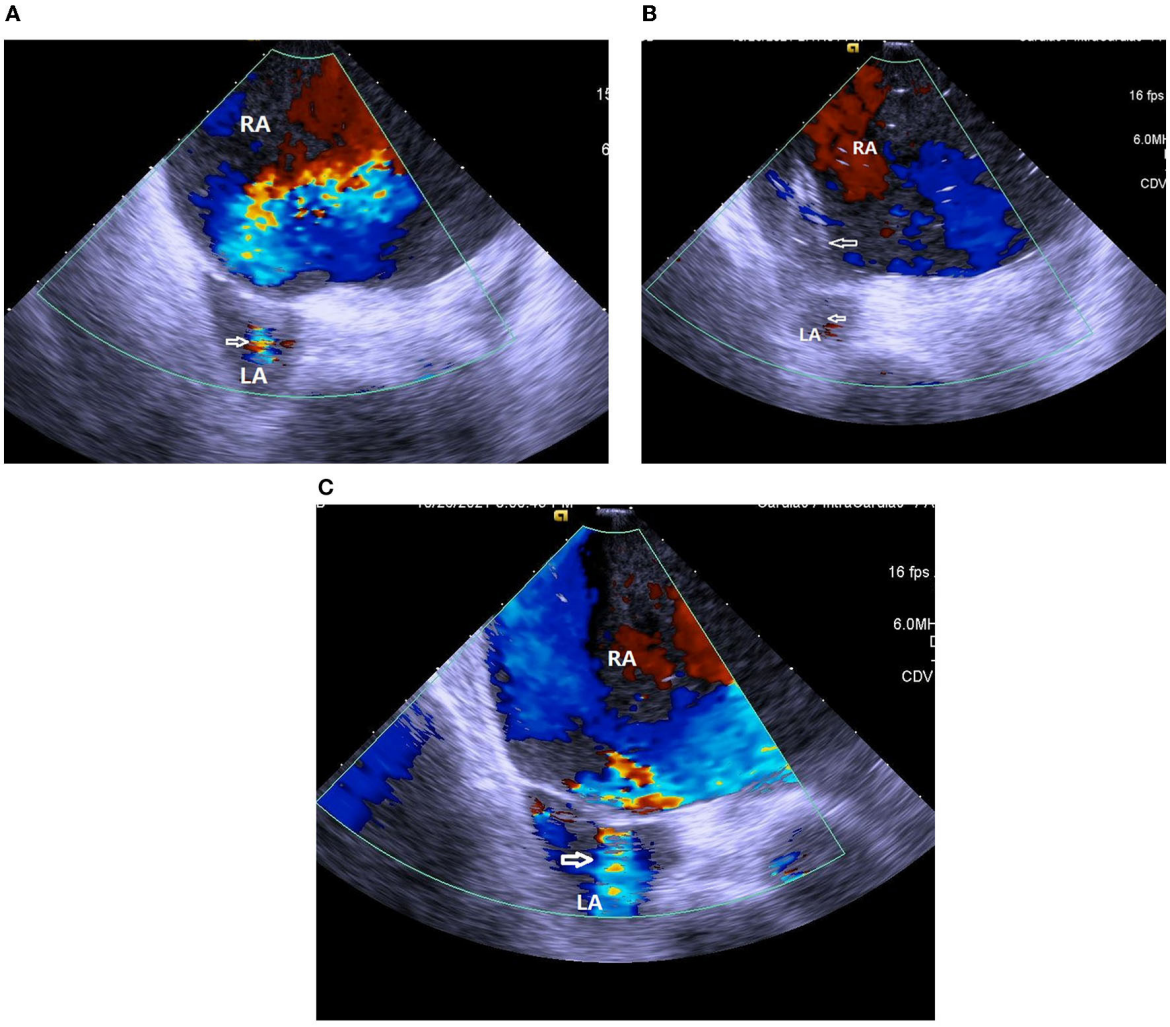
**(A)** ICE confirmed the presence of interatrial shunting (white arrow) created by previous balloon atrial septostomy. RA, right atrium; LA, left atrium. **(B)** ICE showed inflation of an 8 mm balloon (white arrows) at the second session of balloon atrial septostomy. RA, right atrium; LA, left atrium. **(C)** ICE showed an increase of right to left shunting (white arrow) after second session of balloon atrial septostomy. RA, right atrium; LA, left atrium.

A 37-year-old woman with IPAH had received maximal target medications including oral phosphodiesterase type-5 inhibitor, endothelin receptor antagonist, and subcutaneous PA (treprostinil) injection. Other than IPAH, she had no remarkable past medical history. In April 2020, she developed worsening right heart failure with peripheral edema, oliguria, and hypotension (1st index admission). Upon admission, her systolic blood pressure was 92/76 mmHg, pulse rate was 107 beats/min, pulse oximetry was 91% in room air, and respiratory rate 20/min. Physical examinations revealed a jugular venous giant V wave with estimated central venous pressure ≥ 20 cmH_2_O, a grade II pansystolic murmur, and a palpable heave at the left lower sternal border. Laboratory data showed anemia with hemoglobin 7.8 g/dL, hypokalemia 3.0 mEq/L, and elevated NT-pro BNP of 4,138 g/dL. Trans-thoracic echocardiogram revealed a marked dilated RA and right ventricle (RV), in addition to a small and compressed left atrium (LA) and left ventricle (LV) (Figure 1A). She had a transition of subcutaneous to IV PA injection via a peripherally inserted central catheter (PICC) and was registered for a lung transplantation waiting list. On the 7th day of admission, we performed standard right and left heart catheterization. The baseline mean RA pressure was 14 mmHg, aortic oximetry was 96% under oxygen supply at 3 L/min through a nasal cannula, systemic CO was 2.9 L/min, and systemic OD was 291 cc/min (Table 1). An intracardiac echocardiogram (ICE) catheter (AcuNac catheter, Siemens, Mountain View, CA, USA) was introduced via the left femoral vein into RA, and the image was displayed on an ACUSON SC 2000 System (Siemens, Mountain View, CA, USA). Using real-time ICE guidance, the inter-atrial septum and fossa ovalis were clearly visualized. We used a transseptal Brockenbrough needle and a Mullins sheath (Medtronic, Minneapolis, MN, USA) to probe the inter-atrial septum before entering the LA cavity under ICE guidance (Figure 1B). The atrial septum was subsequently dilated with a 5 mm × 8 cm and 6 mm × 8 cm Mustang balloon (Boston Scientific, MA, USA) (Figure 1C). After BAS, systemic CO was increased to 3.5 L/min and OD to 318 mL/min, while the systemic arterial oxygen saturation was dropped down to 87% (under nasal cannula 3 L/min oxygen supply) (Table 1). However, we observed no immediate fall of the RA pressure (Table 1). Transthoracic echocardiogram confirmed the establishment of an inter-atrial shunting from right to left (Figure 1D). After BAS in conjunction with continuous IV PA infusion, her heart failure symptoms were alleviated to function II. Her symptoms remained stable thereafter. One and a half years later, she was re-admitted for changing the PICC and to receive cardiac catheterization again as well. ICE confirmed the presence of an interatrial shunt created by previous BAS (Figure 2A). The second baseline hemodynamic data revealed mean RA pressure of 5 mmHg, LA pressure of 2 mmHg, and systemic CO of 3.3 L/min and OD of 403 mL/min. The findings were in support of the sustained hemodynamic improvements (Table 1). The second course of BAS was done with an 8 mm balloon (Figure 2B). ICE showed increased shunting from right to left after the second BAS (Figure 2C). Post second BAS catheterization measurements revealed mean RA pressure of 3 mmHg, LA pressure of 3 mmHg, systemic CO 3.4 L/min, and OD of 420 cc/min (Table 1). She was discharged with stable functional II symptoms.

**Table 1:** Hemodynamic parameters before and after balloon atrial septostomy in a patient with idiopathic pulmonary arterial hypertension.

	**Baseline (May 5, 2020)**	**Post-1st BAS (May 5, 2020)**	**2nd Baseline (Oct. 26, 2021)**	**Post 2nd BAS (Oct. 26, 2021)**
Arterial O_2_ saturation (%) under O_2_ 3 L/min	96%	87%	95%	96%
PA pressure (s/d/m, mmHg)	79/39/56	87/45/61	59/37/47	57/35/45
PVR (Wood unit)	17.5	19.2	15	14
Systemic cardiac output (L/min)	2.9	3.5	3.3	3.4
Qp/Qs	1	0.85	0.9	0.9
Hemoglobin (g/dL)	7.8	7.8	9.6	9.6
Systemic oxygen delivery (ml/min)	291	318	403	420
RA pressure (mean, mmHg)	14	14	5	3
LA pressure (mmHg)	6	4	2	3

*BAS, balloon atrial septostomy; LA, left atrium, PA, pulmonary artery; PVR, pulmonary vascular resistance; Qp/Qs, pulmonary blood flow/systemic blood flow; RA, right atrium*.

## Discussion

For IPAH with advanced right heart failure, the prognosis is poor (1). Regarding treatment options, continuous IV PA infusion is indicated (1). BAS creates a right-to-left inter-atrial shunt which decompresses the right heart and increases the preload of the left heart (4, 5). At the expense of reducing systemic arterial oxygen saturation, the systemic CO and OD are increased (4, 5). Case reports, case series, and meta-analysis reported immediate hemodynamic benefits of BAS (3–5). Fewer reports were published on the mid-term hemodynamic benefits of BAS (6). Our present case study found sustained hemodynamic improvements, including lower RA pressure and higher CO and OD, and also a sustained functional recovery to II at 1.5 years after the treatments of BAS and IV PA. This corroborates that BAS is a bridging treatment option before lung transplantation becomes available.

From the time of disease diagnosis of pulmonary arterial hypertension, the 5-year survival rate is very poor (57%) as revealed by historical data from the Registry to Evaluate Early and Long-term PAH Disease Management (REVEAL registry) (7). Subjects with IPAH and advanced right heart failure admitted to the intensive care unit (ICU) have a 41% mortality rate (1, 8). Chiu et al. (9) reported that patients with severe PAH receiving BAS had a 66% of 1-year transplant-free survival. Since there is no randomized control trial demonstrating survival benefits of BAS, the role of BAS in IPAH is limited and can serve as a bridging option before lung transplantation. This case showed a possible mid-term survival benefit conferred by BAS and IV PA infusion for a patient with IPAH who had been admitted to ICU due to advanced right heart failure while on a queue list for lung transplantation.

For this patient, we added procedural refinements to improve the safety and efficacy of BAS. First, we followed the existing guidelines and literature to perform a graded septum dilatation (1, 6, 10). Our patient received 5, 6 mm balloon dilatation in the first BAS session and 7, 8 mm balloon dilatation in the second BAS session at an inter-session interval of 1.5 years. Second, the current guidelines and literature suggest avoiding very high-risk scenarios, such as mean RA pressure > 20 mmHg or room air pulse oximetry <85% (3, 6, 10). The patient was treated in line with the current recommendations. Third, we used ICE to guide the interatrial septum puncture, providing an instant clear view of the septum tenting sign (Figure 1B) and sparing the need for transesophageal echocardiogram and tracheal intubation (4, 5).

The cause of anemia in this patient could be multi-factorial. The first was a possible common side effect of fluid retention by endothelin receptor blocker (11). The second could be iron deficiency by menstruation blood loss. Her serum iron was low, around 20 μg/dl. She had received ferrous supplementation since 2017. Blood transfusion was done in the 1st index admission. The improvement of systemic oxygen delivery was in part due to correction of anemia.

We concluded that BAS and IV PA infusion are effective in maintaining mid-term hemodynamic benefits and stabilizing the critical right heart failure in IPAH patients over a 1.5-year interval. Such treatment is therefore a therapeutic option before lung transplantation.

## Data Availability Statement

The original contributions presented in the study are included in the article/supplementary material, further inquiries can be directed to the corresponding author/s.

## Ethics Statement

The patient signed informed consent for the publication of this report.

## Author Contributions

All authors listed have made a substantial, direct, and intellectual contribution to the work and approved it for publication.

## Conflict of Interest

The authors declare that the research was conducted in the absence of any commercial or financial relationships that could be construed as a potential conflict of interest.

## Publisher's Note

All claims expressed in this article are solely those of the authors and do not necessarily represent those of their affiliated organizations, or those of the publisher, the editors and the reviewers. Any product that may be evaluated in this article, or claim that may be made by its manufacturer, is not guaranteed or endorsed by the publisher.
